# The Mechanism of Melatonin and Its Receptor MT2 Involved in the Development of Bovine Granulosa Cells

**DOI:** 10.3390/ijms19072028

**Published:** 2018-07-12

**Authors:** Shujuan Wang, Wenju Liu, Xunsheng Pang, Sifa Dai, Guodong Liu

**Affiliations:** 1 College of Animal Science, Anhui Science and Technology University, Fengyang 233100, China; wangsj@ahstu.edu.cn (S.W.); pangxs@ahstu.edu.cn (X.P.); daisf@ahstu.edu.cn (S.D.); 2 Research Center for Biomedical and Health Science, Anhui Science and Technology University, Fengyang 233100, China

**Keywords:** RNA interference, shRNA, melatonin, granulosa cell, MT2, hormone, gene regulation

## Abstract

Ovarian granulosa cells (GCs) are a critical approach to investigate the mechanism of gene regulation during folliculogenesis. The objective of this study was to investigate the role of *MT2* in bovine GCs, and assess whether *MT2* silencing affected GCs response to melatonin. We found that *MT2* silencing significantly decreased the secretion of progesterone and estradiol, and increased the concentration of inhibin B and activin B. To further reveal the regulatory mechanism of *MT2* silencing on steroids synthesis, it was found that the expression of *CYP19A1* and *CYP11A1* enzymes (steroid hormone synthesis) were down-regulated, while genes related to hormonal synthesis (*StAR*, *RUNX2*, *INHA* and *INHBB*) were up-regulated without affecting the expression of *INHBA*, suggesting that *MT2* silencing may regulate hormone abundance. Furthermore, *MT2* silencing significantly increased the expression of *TGFBR3* and *BMP6*, and decreased the expression of *LHR* and *DNMT1A* without significant difference in the expression of *FSHR* and *EGFR*. In addition, *MT2* silencing didn’t affect the effect of melatonin on increasing the expression of *DNMT1A*, *EGFR*, *INHBA* and *LHR*, and progesterone level, or decreasing *INHA*, *TGFBR3* and *StAR* expression, and production of inhibin B. Moreover, *MT2* silencing could disrupt the role of melatonin in decreasing the *FSHR*, *INHBB* and *BMP6* expression, and activin B secretion. In conclusion, these results reveal that melatonin and MT2 are essential regulator of bovine GCs function by modulating reproduction-related genes expression, hormones secretion and other regulators of folliculogenesis.

## 1. Introduction

Ovarian folliculogenesis is a complicated process and it occurs from the primordial follicles before or immediately after birth [1]. During the estrous cycle, the folliculogenesis mainly include the following process: primordial follicles are activated to grow into primary follicles and subsequently into secondary follicles; antral follicles are then formed, and further developed antral follicles are finally ovulated [2]. However, more than 99% of follicles undergo atresia, and less than 1% are selected for ovulation [3]. Many factors are involved in the ovarian folliculogenesis process, such as endocrine and local autocrine/paracrine systems, as well as the factors produced by granulosa cells [4,5,6,7,8]. Follicular development is accompanied by the proliferation, cell cycle control and functional differentiation of GCs [8,9]. Meanwhile, GCs apoptosis could initiate the follicular atresia [2,10,11]. Therefore, ovarian GCs act as an important physiological role in regulating the ovarian follicular development. Moreover, GCs are useful models for investigating the molecular mechanisms of gene regulation during folliculogenesis [9]. It is of particular interest in investigating the mechanisms of follicular development and atresia caused by GCs.

It is well-known that luteinizing hormone (LH) and follicle stimulating hormone (FSH) bind to their receptor LHR and FSHR to further regulate the follicle growth. Apart from the above hormones, the steroid hormones (including estradiol and progesterone), members of TGF-β (transforming growth factor-β) super family (including activins and inhibins) and other factors secretion by GCs are also critical for maintenance of the ovarian cycle [12]. Progesterone is important not only for luteinization and maintenance of pregnancy, but also as a substrate for most other steroids [13]. The abundance of progesterone is mediated by the expression of steroidogenic acute regulatory protein (StAR) and cytochrome P450 cholesterol side-chain cleavage enzyme (P450scc, encoded by *CYP11A1*). StAR is involved in the rate limiting step in steroidogenesis and *CYP11A1* encodes the enzyme taking part in the first step of the steroidogenesis and convertes cholesterol to pregnenolone, respectively [13,14,15]. Apart from cholesterol, 7-dehydrocholesterol and vitamins D3 are also identified as substrates for *CYP11A1* [16,17,18].

During follicle growth GCs produce increasingly more of oestradiol and the *CYP19A1* encodes the rate limiting step for oestradiol synthesis [15]. Moreover, the level of estradiol is related to follicles maturation, with higher production secreted by GCs in dominant follicles than those in subordinate follicles [19,20]. In addition, inhibins suppress the further development of subordinate follicles through decreasing FSH secretion, and act as a negative feedback on the hypothalamic-pituitary system. 

Beside the hormones and factors mentioned above, melatonin is another more interesting in regulating the hypothalamic-pituitary-gonadal axis in mammalian. It is well-known that melatonin plays important role in modulating ovarian function. For example, melatonin is involved in follicle development, inducing oocyte maturation, protecting GCs function, mediating hormone secretion of GCs, as well as promoting the embryos development [21,22,23,24,25,26,27,28]. The pineal gland is not the only source of melatonin, and GCs and mitochondria can also produce the melatonin [22,29,30,31]. Moreover, high concentration of melatonin in follicular fluid indicates that melatonin acts as a direct role in folliculogenesis [22,23,32]. Although the factors secreted by GCs play important role in maintenance of ovarian function and are essential for their survival, the role of melatonin and its receptor MT2 is little known in mediating the GCs function. 

Our previous study found that MT2 was involved in regulating the GCs apoptosis and the apoptosis related genes expression. Moreover, *MT2* knockdown didn’t affect the effects of melatonin on suppressing the GCs apoptosis or blocking the cell cycle [33]. In this study, we revealed the role of MT2 in modulating reproduction-related genes expression, hormones secretion and other regulators of folliculogenesis using *MT2* silencing. To further evaluate the regulatory mechanism of MT2 on bovine GCs, we analyzed the expression of genes related to reproduction (*FSHR* and *LHR*), hormone (*StAR*, *CYP19A1* [Cytochrome P450, family 19, subfamily A, polypeptide 1], *CYP11A1* [Cytochrome P450, family 11, subfamily A, polypeptide 1], *RUNX2* [Runt related transcription factor 2], *INHA* [Inhibin alpha], *INHBA* [Inhibin beta A subunit] and *INHBB* [Inhibin beta B subunit]), and development (*DNMT1A* [DNA methyltransferase 1a], *EGFR* [Epidermal growth factor receptor], *TGFBR3* [Transforming growth factor beta receptor 3] and *BMP6* [Bone morphogenetic protein 6]). Furthermore, the response of GCs to melatonin treatment after *MT2* knockdown on related genes expression and hormone secretion were also studied to reveal the effects of melatonin-MT2 signaling on folliculogenesis in bovine GCs. The present study helped to understand the role of MT2 in modulating GCs functions and also provided important insights on the mechanisms by which melatonin signaling affects these functions.

## 2. Results

### 2.1. pshRNA-MT2 Efficiently Silenced MT2 Expression in Bovine GCs

**Figure 1:**
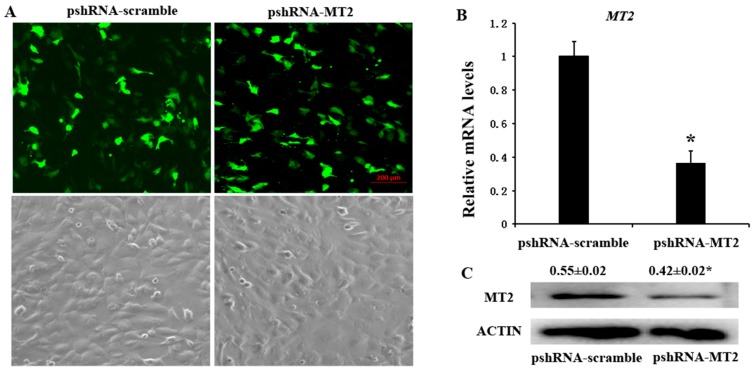
Identification the silencing efficiency at 48 h after transfection with pshRNA-MT2 in bovine GCs. (**A**) The expression of GFP indicated that pshRNA-MT2 and pshRNA-scramble were efficiently transfected in GCs; (**B**,**C**) *MT2* mRNA (**B**) and protein level (**C**) were measured after 48 h transfected with pshRNA-MT2 using real-time PCR and western blotting, respectively. pshRNA-MT2 was able to silence the *MT2* mRNA and protein level. Results are present as the mean ± SEM. Statistical analyses were performed using one-way ANOVA (analysis of variance) with Duncan’s test: *p* < 0.05 (*). The experiment was repeated three times independently.

pshRNA-MT2 and pshRNA-scramble plasmids were transfected into GCs, when the GCs reach 70–80% confluence at the time of transfection. The GFP (green fluorescent protein) was used to monitor the transfection efficiency beginning from 24 h after transfection (Figure 1A), and the silencing efficiency was finally confirmed by measuring the *MT2* mRNA and protein abundances (Figures S1 and S2). The results showed that pshRNA-MT2 could effectively silence the mRNA and protein abundances of MT2 compared to pshRNA-scramble in GCs (Figure 1B,C).

### 2.2. Effects of MT2 Silencing and Melatonin Treatment on Reproduction Related Genes Expression

**Figure 2:**
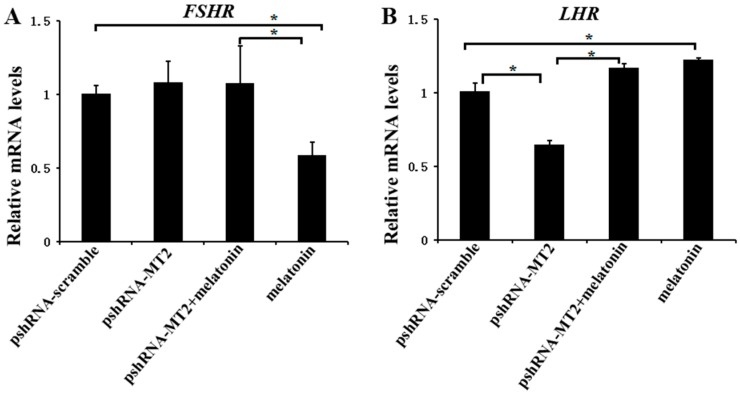
Effects of *MT2* gene silencing and melatonin treatment on the expression of reproduction related genes (*FSHR* and *LHR*). (**A**,**B**) The mRNA levels of *FSHR* (**A**) and *LHR* (**B**) were measured by real-time PCR in GCs at 48 h after transfection with pshRNA-MT2 and/or melatonin treatment. The quantity of mRNA was normalized to that of *ACTB*. Results are present as the mean ± SEM. The Statistical analyses were performed using one-way (ANOVA) (analysis of variance) with Duncan’s test: *p* < 0.05 (*). The experiment was repeated three times independently.

To determine whether MT2 and melatonin-MT2 signaling were involved in the modulation of FSH and LH receptor, we measured the expression of *FSHR* and *LHR* after *MT2* silencing and melatonin treatment (Figure 2; Supplementary Materials). *MT2* silencing didn’t change the expression of *FSHR* (*p* > 0.5), however, the expression of *LHR* was significantly decreased after *MT2* silencing compared to the pshRNA-scramble group (*p* < 0.5). In addition, *MT2* silencing disturbed the effect of meltonin on suppressing *FSHR* expression (Figure 2A, *p* < 0.5). As for *LHR*, melatonin promoted its expression dramatically either in presence or in absence of *MT2* silencing in GCs (Figure 2B, *p* < 0.5).

### 2.3. Effects of MT2 Silencing and Melatonin Treatment on Endocrine Secretions and Endocrine Related Genes Expression

**Figure 3:**
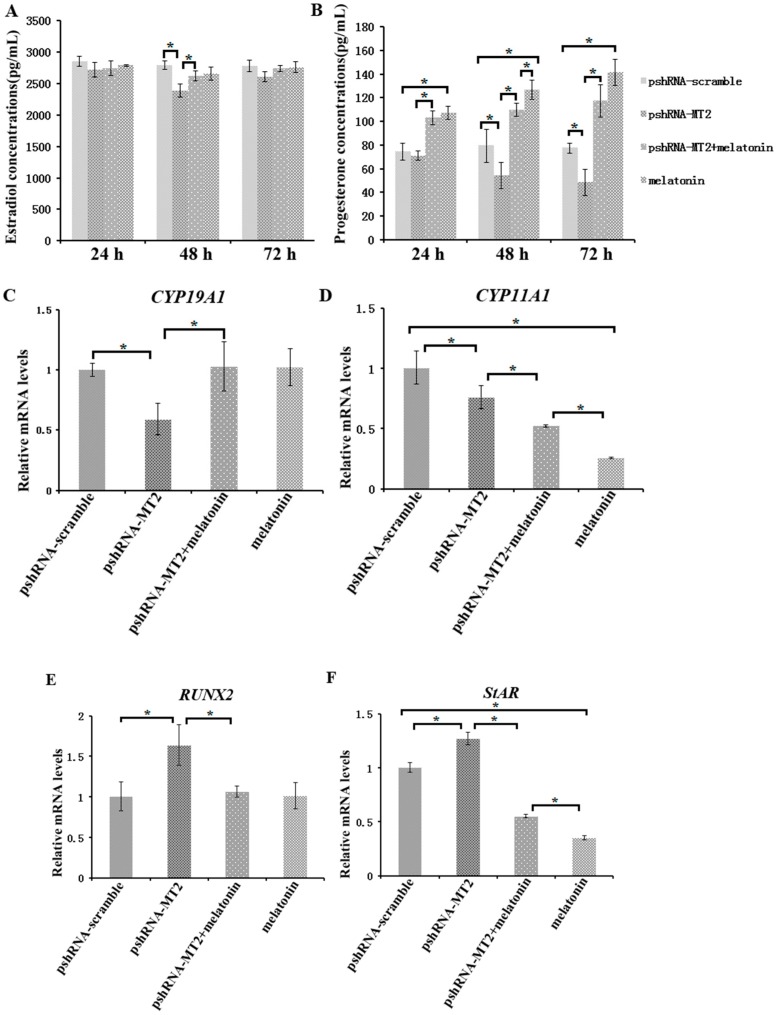
Effect of *MT2* silencing and melatonin treatment on endocrine secretions (progesterone and estradiol) and endocrine related genes expression (*StAR*, *CYP19A1*, *CYP11A1* and *RUNX2*). (**A**,**B**) Levels of estradiol (**A**) and progesterone (**B**) were measured at 24, 48, and 72 h in GCs medium after transfection with pshRNA-MT2 and/or melatonin treatment. (**C**–**F**) The mRNA abundance of *CYP19A1* (**C**), *CYP11A* (**D**), *RUNX2* (**E**) and *StAR* (**F**) were measured by real-time PCR at 48 h after transfection with pshRNA-MT2 and/or Melatonin treatment. mRNA abundance was normalized to that of *ACTB*. Results are present as the mean ± SEM. Statistical analyses were performed using one-way ANOVA with Duncan’s test: *p* < 0.05 (*). The experiment was repeated three times independently.

**Figure 4:**
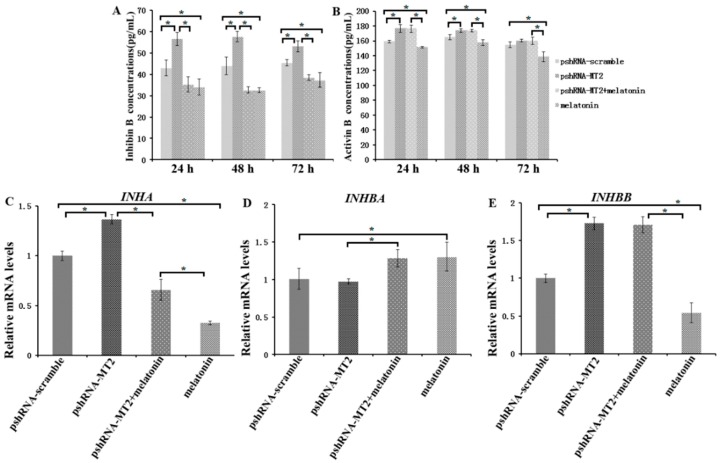
Effect of *MT2* silencing and melatonin treatment on endocrine secretions (inhibin B and activin B) and endocrine related genes expression (*INHA*, *INHBA* and *INHBB*). (**A**,**B**) Abundance of inhibin B (**A**) and activin B (**B**) were measured at 24, 48, and 72 h in GCs medium after transfection with pshRNA-MT2 and/or melatonin treatment. (**C**–**E**) The mRNA abundance of *INHA* (**C**), *INHBA* (**D**) and *INHBB* (**E**) were examined by real-time PCR at 48 h after transfection with pshRNA-MT2 and/or melatonin treatment. mRNA abundance was normalized to that of *ACTB*. Results are present as the mean ± SEM. Statistical analyses were performed using one-way ANOVA with Duncan’s test: *p* < 0.05 (*). The experiment was repeated three times independently.

To assess the role of *MT2* silencing on endocrine secretions, we detected the abundance of estradiol, progesterone, inhibin B and activin B in culture medium at 24 h, 48 h and 72 h after *MT2* silencing and melatonin treatment (Figure 3 and Figure 4; Supplementary materials). The results indicated the concentration of progesterone and estradiol was significant lower in pshRNA-MT2 group than that in pshRNA-scramble group after transfection (Figure 3A,B, *p* < 0.5). In addition, the release of inhibin B and activin B was significant higher in pshRNA-MT2 group compared to that of pshRNA-scramble group (Figure 4A,B, *p* < 0.5). Moreover, after melatonin treatment, the effect of suppressing progesterone and promoting inhibin B secretion was reversed and rescued so that progesterone concentration was higher and inhibin B level was lower in GCs, regardless of whether or not *MT2* was silenced compared to pshRNA-MT2 and pshRNA-scramble group (Figure 3B and Figure 4A, *p* < 0.5). While estradiol concentration was not affected after melatonin treatment compared to pshRNA-scramble group (Figure 3A, *p* > 0.5). Conversely, *MT2* silencing interrupted the role of melatonin in suppressing the secretion of activin B (Figure 4B, *p* < 0.05).

To further confirm the effects of *MT2* silencing and melatonin treatment on endocrine secretions, we analyzed the hormone related genes (*StAR*, *CYP19A1*, *CYP11A1*, *RUNX2*, *INHA*, *INHBA* and *INHBB*) expression by real-time PCR (Supplementary materials). The results showed that *MT2* silencing significantly downregulated the expression of *CYP19A1*, which encodes the rate limiting step for oestradiol synthesis, while it significantly upregulated *RUNX2* expression, and there were no significant difference in the expression of *CYP19A1* and *RUNX2* after melatonin treatment compared to the pshRNA-scramble group (Figure 3C,E). Both *CYP11A1* and *StAR* are important regulator of progesterone synthesis. It was found the expression level of *CYP11A1* was decreased and the expression level of *StAR* was increased after *MT2* silencing, and melatonin significantly decreased their expression with or without *MT2* silencing compared to that in pshRNA-MT2 and pshRNA-scramble group in the GCs (Figure 3D,F, *p* < 0.05). Moreover, *INHA* and *INHBB* were significantly upregulated after *MT2* silencing compared to the pshRNA-scramble group (Figure 4C,E, *p* < 0.05). Conversely, melatonin treatment significantly downregulated the expression of *INHA* compared to the pshRNA-scramble group, even if *MT2* was silenced (Figure 4C, *p* < 0.05). However, *MT2* silencing affected the effect of melatonin on suppressing the expression of *INHBB* (Figure 4E, *p* < 0.05). In addition, no change was observed in the expression of *INHBA* after *MT2* silencing, and its expression was significantly upregulated after melatonin treatment in the pshRNA-MT2+melatonin and melatonin group compared to pshRNA-MT2 and the pshRNA-scramble group (Figure 4D). The above results indicate melatonin-MT2 signaling was involved in modulating of endocrine secretions in bovine GCs.

### 2.4. Effects of MT2 Silencing and Melatonin Treatment on Development Related Genes Expression

**Figure 5:**
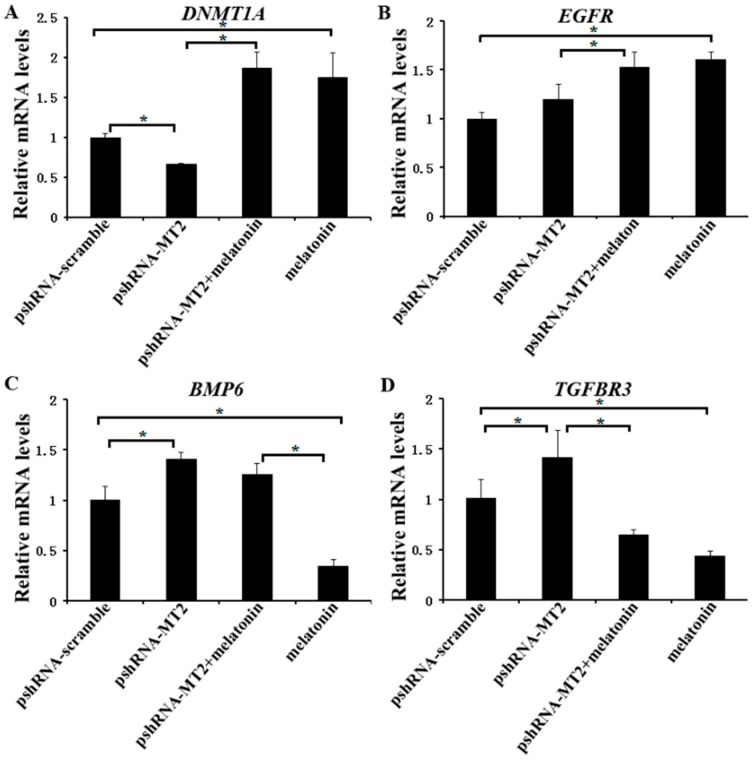
Effect of *MT2* silencing and melatonin treatment on the expression of development (*DNMT1A*, *EGFR*, *TGFBR3* and *BMP6*) in GCs. (**A**–**D**) The expression of *DNMT1A* (**A**), *EGFR* (**B**), *BMP6* (**C**) and *TGFBR3* (**D**) were examined by real-time PCR in GCs at 48 h after transfection with pshRNA-MT2 and/or melatonin treatment. The quantity of mRNA was normalized to that of *ACTB*. Results are present as the mean ± SEM. Statistical analyses were performed using one-way ANOVA with Duncan’s test: *p* < 0.05 (*). The experiment was repeated three times independently.

We further investigated the expression of *DNMT1A*, *EGFR*, *TGFBR3* and *BMP6* (Supplementary Materials), which are related to the folliculogenesis, to elucidate their potential role of melatonin and *MT2* in bovine ovary. The results indicated that *MT2* silencing significantly reduced the mRNA level of *DNMT1A*, but increased the mRNA level of *TGFBR3* and *BMP6.* There was no significant difference in the expression of *EGFR* compared to the pshRNA-scramble group (Figure 5A–D, *p* < 0.05). Moreover, melatonin significantly increased the mRNA level of *DNMT1A* and *EGFR*, and reduced the mRNA level of *TGFBR3* either in the presence or absence of *MT2* silencing in GCs (Figure 5A,C,D, *p* < 0.05). With regard to *BMP6*, *MT2* silencing interrupted the effect of melatonin on decreasing its mRNA level (Figure 5B, *p* < 0.05). These results suggested that melatonin and MT2 played important role in folliculogenesis.

## 3. Discussion

Unluteinized GCs play an important role in modulating the ovarian follicular development, such as folliculogenesis, follicle selection, oocyte maturation, follicular atresia, and also act as an important role in regulating the ovary function by local autocrine/paracrine systems. Our previous studies have demonstrated that melatonin was involved in modulating the GCs functions [25,27,33]. However, there is little information about the role of MT2 in modifying the reproduction related genes**,** hormone related genes, development related genes and hormone secretion, as well as whether MT2 affects the GCs response to melatonin. Therefore, we investigated the reproduction related function of MT2 and melatonin-MT2 signaling in this study by *MT2* silencing and melatonin treatment in bovine GCs.

The hormone secretion by hypothalamic-pituitary, such as FSH and LH, plays important role in maintaining the follicle viability, inducing the follicle growth and luteinization via binding to their receptor FSHR and LHR in the ovary [22,23,34,35,36,37]. FSH can act as a regulating role in GCs from primary follicle stages onward via binding to its receptor FSHR [38]. FSHR may be directly involved in the initial growth of primordial follicles in caprine [39]. Moreover, it is verified that melatonin alone or in association with FSH promote in vitro development of isolated caprine secondary follicles [39]. Melatonin has exhibited stimulatory action on LH secretion during the luteal phase of the estrous cycle in ewes and enhancement follicular response to LH through upregulating *LHR* expression [24,36,40]. Therefore, melatonin is involved in modulating the function of FSH and LH via affecting their receptors expression in some physiological stages. The results in this study showed that *MT2* silencing significantly downregulated *LHR* expression and didn’t change the expression of *FSHR*. Moreover, *MT2* silencing changed the effect of melatonin on downregulating *FSHR* expression, while didn’t affect the GCs respond to melatonin on *LHR* expression. Melatonin-MT2 signaling was also involved in enhancing LH action by elevating *LHR* expression. Similarly, the higher concentration of melatonin in serum could improve corpus luteal function and then have high pregnancy rate in bovine [37].

An important aspect of the present research is to investigate the responses of GCs to *MT2* silencing on the secretion of progesterone and estradiol, as well as other regulatory factors produced by GCs. The concentration of progesterone and estradiol decreased significantly at 48h after *MT2* silencing compared to the pshRNA-scramble group in GCs culture system. Melatonin treatment significantly increased the level of progesterone, while didn’t change the level of estradiol in GCs either in presence or absence of *MT2* silencing. However, the reduction in the expression of *CYP19A1* and *CYP11A1* were observed after *MT2* silencing in the GCs, which are involved in the steroid hormone synthesis [13,15,41]. Therefore, *MT2* silencing modulated the transcription of the steroidogenic enzymes, and then affected the secretion of steroid hormone. Curiously, contrary to promotion in progesterone level, we observed that the expression of *CYP11A* and *StAR* was significantly inhibited by melatonin treatment both in presence or absence of *MT2* silencing in GCs. However, transcriptional level of *StAR* increased significantly after silencing of *MT2* in GCs. Consistent with the present study, in response to lower levels of progesterone, the expression of *CYP11A1* was inhibited and *StAR* had an increasing expression pattern, and melatonin inhibited the expression of *CYP11A*, despite an increase in progesterone production [9,42]. We presume that the high levels of progesterone secreted by GCs act as a negative feedback regulator of transcription of *CYP11A* and *StAR* after melatonin treatment [9,41,42,43,44,45]. *RUNX2* is another important transcription factor in modulating ovulation and/or luteal development [46]. The results in this study indicated that *MT2* silencing significantly upregulated the *RUNX2* expression and downregulated the estradiol level, which is consistent with the previous study that the expression of *RUNX2* is negatively associated with estradiol abundance in human ovary and in porcine GCs [9,47]. The present results elucidated that melatonin-MT2 signaling acted as a direct regulatory action on steroidogenesis via mediating the steroid hormone synthesis related genes *CYP19A1*, *CYP11A1*, *StAR* and *RUNX2*.

The BMPs as autocrine/paracrine factors act a functional roles in mammals ovary function [48,49]. In the present study, we also analyzed the role of *MT2* silencing and melatonin treatment in modulating the expression of *BMP6*. The result indicated that *MT2* silencing significantly increased *BMP6* expression. In contrary, melatonin treatment significantly suppressed the expression of *BMP6* in the melatonin group. Moreover, *MT2* silencing disturbed the effect of melatonin on suppressing the expression of *BMP6*. BMP6 is well-known for its inhibition FSH action by suppressing adenylate cyclase activity downstream of the FSHR in GCs, and involved in the selection of dominant follicles and inhibitor of luteinization [50,51,52]. Recent studies show that melatonin reverses the inhibitory effect of BMP-6 on FSH-induced progesterone production, and importantly, melatonin impaired the inhibitory effects of BMP-6 on the steroidogenetic enzyme expression in GCs [48,52,53]. Furthermore, the further study finds that melatonin plays a key role in regulation of BMP-6 signal intensity and thus controls the progesterone production in the ovary [52]. These findings suggest that melatonin-MT2 signaling play important role in maintenance of ovarian function through regulating the BMP6 activity in GCs.

There is an accumulation of findings regarding the TGF-β superfamily regulating the follicular development. The inhibins and activins produced by GCs are involved in control of follicle growth through a complex biological process including interdependent endocrine, autocrine and paracrine system [54,55,56]. The inhibin acts a classical negative feedback mechanism for secretion of FSH and estradiol by granulosa cells [54,57]. In contrary, activin promotes the secretion of FSH, and is an enhancer of *FSHR* expression, as well as decreases progesterone production in human GCs [56,58]. In the present study, we found that *MT2* silencing significantly increased the levels of inhibin B and activin B, and melatonin reversed the promotion effect on inhibin B secretion caused by *MT2* silencing. However, *MT2* silencing affected the effect of melatonin on suppressing the secretion of activin B. We also further elucidated the expression of hormone related genes after *MT2* silencing. In accordance with change of inhibin B and activin B, *MT2* silencing significantly upregulated the expression of *INHA*, *INHBB* and *TGFBR3*, and didn’t change the *INHBA* level. In addition, *MT2* silencing didn’t affect melatonin downregulating the expression of *INHA* and *TGFBR3*, and upregulating the expression of *INHBA* in GCs. However, *MT2* silencing could affect the effect of melatonin on reducing the expression of *INHBB*. Similarly, Inha reduction accompanies by a decrease in the expression of *TGFBR3*, and thus promotes the FSH abundance in the rat anterior pituitary cells, which may be caused by disrupting the inhibin-TGFBR3 signaling [59]. In order to attenuate the inhibition effect on FSH activity, the inhibin vaccines has been investigated in different animals, which indicate that fertility and ovulation rates are improved in cattle, sheep and rats [60,61,62]. Taken together, melatonin-MT2 signaling modulated the inhibin and activin level, and thus was involved in regulating follicular development.

The progress of follicular development is modulated by many genes, and here we assessed the regulatory function of melatonin and MT2 in the expression of *DNMT1A* and *EGFR* in GCs. *MT2* silencing significantly reduced the level of *DNMT1A* and there was no difference in the expression of *EGFR* after *MT2* silencing. Moreover, *MT2* silencing didn’t alter the enhancing the action of melatonin on both the expression of *DNMT1A* and *EGFR*. Consistent with the present study, melatonin significantly upregulated the expression of *DNMT1A* and *EGFR* involved in signal transduction and epigenetic reprogramming during bovine oocyte maturation [24]. In addition, the same effect was observed on the expression of *DNMT1A* in the bovine blastocyst and sheep oocyte and EGFR in sheep cumulus cell after melatonin treatment [63,64]. EGFR is crucial for mediating the role of EGF-like proteins, which act as a crucial role in modulating the action of LH, and thus regulate oocytes maturation and ovulation [65]. DNMT1A is involved in DNA methylation status, and then regulates gene expression [66]. In this study, we observed that melatonin and MT2 affected the expression level of *DNMT1A* and *EGFR*; those results suggest that melatonin-MT2 signaling may regulate GCs epigenetic status and enhance EGF-like proteins with elevating LHR level.

Both MT1 and MT2 are expressed on bovine granulosa cells [25]. Some effects of melatonin are mediated through binding to MT1 and MT2 [18], whereas some actions seem to depend on the retinoid-related orphan nuclear hormone receptor family (RZR/ROR) [67,68]. However, recent study indicates that RORα is not a receptor for melatonin [69]. Depending on the tissue, organ, and species, melatonin activates different second messenger cascades by interacting with the same receptor subtype [22]. Although MT1 and MT2 are not the only signal transduction mechanism that melatonin can trigger, and it is difficult to evaluate the melatonin action on granulosa cells by which receptors mediating. However, the role of MT2 and melatonin-MT2 signaling affecting the function of bovine GCs could be investigated by *MT2* silencing. Hence, in the present study, we investigated the participation of MT2 in regulating the function of bovine GCs and tested whether the melatonin-MT2 signaling affects the function of bovine GCs. We found that melatonin and MT2 were essential regulator of bovine GCs function by modulating reproduction-related genes expression, hormones secretion and other regulators of folliculogenesis. Moreover, *MT2* silencing didn’t alter some effects of melatonin in the GCs. Similarly, our recent studies have showed that MTNR1A silencing don’t affect bovine GCs respond to melatonin [27]. Furthermore, MTNR1B silencing didn’t disrupt the effects of melatonin on apoptosis and cell cycle in bovine GCs [33]. It has been reported that MT1 and MT2 could mediate the modulation of complex reproductive mechanisms [21,25,33,37,70,71,72]. Therefore, MT1 and MT2 may act in a complementary way to mediate the melatonin actions on bovine GCs [27,32]. Consistent with this, MT1 and/or MT2 are involved in mediating the melatonin actions on anti-apoptotic effects in spermatozoa [73] and cell life/death balance [74,75].

## 4. Materials and Methods

### 4.1. Bovine GCs Isolation and Culture

GCs collection was performed as our previously described elsewhere [25,27,32]. Bovine ovaries were obtained from Bengbu abattoir (Bengbu, Anhui, China). The cell pellets isolated from 3–6 mm follicles were digested for 5 min using 0.25% trypsin with 0.025% EDTA (Gibco, Grand Island, NY, USA), and then centrifugated at 1500 rpm for 5 min. The pellets were diluted with DMEM (Dulbecco’s Modified Eagle Medium) (Gibco, Grand Island, NY, USA) supplemented with streptomycin (50 µg/mL), penicillin (50 IU/mL) (Pen-Strep, Invitrogen, Carlsbad, CA, USA), plasmocin (25 µg/mL; invivogen, San Diego, USA), and 10% fetal bovine serum (FBS; Hyclone, UT, USA), and then cultured in 12-well plates. The GCs were cultured at 37 °C in an incubator containing 5% CO_2_. In this study, the protocols for the experiment were reviewed and approved by the Institutional Committee on Animal Care and Use at Anhui Science and Technology University, and experiments were repeated three times independently.

### 4.2. Transfection of Recombinant Plasmids into GCs

Our previous study have successfully built MT2 recombinant plasmid and confirmed the short hairpin RNA (the target site: 5′-GCTACTTCCTGGCCTATTTCA-3′) having greatest effective in silencing the bovine MT2 gene mRNA and protein expression [32]. Therefore, the corroborated recombinant plasmid was referenced for the present study. Moreover, a nonsense sequence was chosen to construct recombinant plasmid as a scramble control. Therefore, the recombinant RNAi plasmids were named as pshRNA-MT2 and pshRNA-scramble, respectively (Table 1). The plasmids were transfected into bovine GCs in a 12-well plate (70–80% confluence per well) using LipofectamineTM LTX with PlusTM Reagent (Invitrogen, Carlsbad, CA, USA) according to manufacturer’s protocols. The GCs and medium were harvested at 48 h after transfection and used for RNA and hormone experiments. In addition, the recombinant plasmid containing a pSIREN-RetroQ-ZsGreen Vector, which can express green fluorescent protein (GFP), was used for monitoring the transfection efficiency under a fluorescence microscope after transfection.

### 4.3. RNA Extraction and Real-Time PCR

To assess the expression of target genes in *MT2* knockdown or melatonin treatment GCs, total RNA was isolated from GCs transfected with pshRNA-MT2 or pshRNA-scramble, and treated with melatonin for 48 h using RNAprep pure cell Kit (Tiangen, Beijing, China) according to the manufacturer’s protocols. RNA was digested with RNase-free DNaseI to remove the genomic DNA. The cDNA was synthesized using a RevertAid First Strand cDNA Synthesis Kit (Thermo Scientific, Waltham, MA, USA). The quantitative real-time PCR was carried out using LightCycler 480 SYBR Green I Master Mix (Roche, Penzberg, Germany) according to our previously reported method [26,27,32]. The primer pairs designed for expression analysis were listed in Table 2. A total of 10 μL reaction solution was prepared by mixing 5 μL of LightCycler 480 SYBR Green I Master Mix, 2 μL of reverse transcribed cDNA, 0.5 μM specific primer, and 2 μL of RNase and DNase-free water. Amplification was performed in an LightCycler 480 II Real-Time PCR System (Roche, Mannheim, Germany) as follows: 95 °C for 30 s, followed by 40 cycles of 95 °C for 5 min, annealing at particular temperatures for 20 s, 72 °C for 20 s. The melting curve analysis were performed after real-time PCR reactions to confirm specific PCR product. Expression levels of each target genes were normalized to ACTB in each sample. The date analysis was using the 2^−∆∆*C*T^ method [76].

### 4.4. Western Blot Analysis

Western blot Analysis was performed as previously described elsewhere [27,32]. The total protein was extracted after GCs transfection for 48 h and separated by 12% polyacrylamide gel electrophoresis, and then transferred to polyvinylidene fluoride membrane (Millipore, Bedford, MA, USA). Firstly, the membrane were incubated with rabbit MT2 antibody (1:400, ab203346, Abcam, Cambridge, UK) and mouse monoclonal antibody ACTB (1:1000, SC-47778, Santa Cruz, Dallas, TX, USA). Later, the membrane was incubated with HRP labeled goat anti-rabbit secondary antibody (SC-2054) and goat anti-mouse secondary antibody (SC-2005) (1:5000; Santa Cruz, Dallas, TX, USA), respectively. Finally, membranes was detected using the Clarity Western ECL kit (Bio-Rad Laboratories, Hercules, CA, USA), and scanned using a ChemiDocXRS chemiluminescent imaging system (Bio-Rad, Hercules, CA, USA).

### 4.5. Endocrine Secretions Detection

Culture medium was harvested at 48 h after *MT2* silencing and/or melatonin treatment. The cell culture supernatants were centrifuged at 1000× *g* for 15 min and collected with a sterile tube. The supernatants were frozen at −80 °C until use. The level of progesterone, estradiol, inhibin B, and activin B were detected using bovine ELISA (enzyme-linked immunosorbent assay) kits, according to the manufacturer’s protocols (Shanghai Bogoo Biological Technology Co., Ltd., Shanghai, China). The sensitivity of estradiol, inhibin and activin B kits is 1.0 pg/mL and progesterone is 0.1 ng/mL.

### 4.6. Experimental Design

The modulating effect of MT2 on related genes expression and hormones secretion were studied. We examined whether *MT2* silencing affect the response of GCs to melatonin treatment, either alone or in presence of melatonin. Our previous research indicated that 1200 pg/mL melatonin acted as a beneficial effect on GCs function [25]. To elucidate the effects of melatonin-MT2 signaling on bovine GCs, the cultured GCs were treated with 1200 pg/mL melatonin with or without *MT2* silencing. Experimental groups were divided as following: pshRNA-scramble group; pshRNA-MT2 group; pshRNA-MT2 plus melatonin group; and melatonin group. Furthermore, in these trials, we assessed the expression level of reproduction related genes (FSHR and LHR), hormone related genes (*StAR*, *CYP19A1*, *CYP11A1*, *RUNX2*, *INHA*, *INHBA* and *INHBB*), and development related genes (*DNMT1A*, *EGFR*, *TGFBR3* and *BMP6*). We also measured the concentrations of progesterone, estradiol, inhibin B and activin B at 24 h, 48 h and 72 h after MT2 silencing and melatonin treatment.

### 4.7. Statistical Analysis

All data were presented as Mean ± SEM of triplicate experiments (*n* = 3). The date were analyzed using univariate analysis of variance (ANOVA) following by Duncan’s test with Statistical Analysis Systems (SAS Inc., Cary, NC, USA). *p* < 0.05 was considered significant difference between treatments.

## 5. Conclusions

Based on our present data, we demonstrated the biological actions of melatonin and MT2 in modulating reproduction related genes expression, hormone secretion and other regulators of folliculogenesis. The results show that melatonin and MT2 act as an essential role in regulating hormone level of progesterone, estradiol, inhibin B, and activin B. Furthermore, the promotion effect could be due to mediate the expression of *CYP19A1*, *CYP11A1*, *StAR*, *RUNX2*, *INHA*, *INHBA* and *INHBB* in GCs. The study also demonstrated that melatonin and MT2 regulated the GCs function by virtue of their roles in reproduction related genes (*LHR* and *FSHR*) as well as the development related genes (*DNMT1A*, *EGFR*, *TGFBR3* and *BMP6*). The study benefits to understand the effect of melatonin and its receptor MT2 on modulating physiological functions and these also provide detailed insights on a potential mechanisms of melatonin-MT2 signaling in regulating these functions.

## Figures and Tables

**Table 1:** Target sequences of bovine *MTNR1B*.

Name	Target sequence (5’→3’)	Position on CDs
pshRNA-MT2 *	GCTACTTCCTGGCCTATTTCA	878
pshRNA-scramble *	CTTCATAAGGCGCATAGC	

* Liu et al [33].

**Table 2:** Sequences of primer pairs for quantitative real-time PCR.

Gene	Forward Primer Sequence (5′→3′)	Reverse Primer Sequence (5′→3′)	Length
*MTNR1B*	GGAGCTTTCTGAGCATGTTTG	CCCTGCGGAAGTTCTGGTT	210
*CYP11A1*	ATGCTGGAGGAGACAGTGAACC	GCAGTAGAGGATGCCTGGGTAA	249
*CYP19A1*	CACCCATCTTTGCCAGGTAGTC	ACCCACAGGAGGTAAGCCTATAAA	78
*StAR*	GTG GAT TTT GCC AAT CAC CT	TTATTG AAA ACG TGC CAC CA	203
*RUNX2*	AAGGCAAGGCTAGGTGGAAT	AGAGGGGCACAGACTTTGAA	189
*DNMT1A*	ACGAATGGTGGATTGCTGGT	CACGTCTTCGTAGGTGGAGTC	197
*EGFR*	CACTCATGCTCTATGACCCTACC	CTCACCGATTCCTATTCCGTTAC	176
*BMP6*	TACGCTGCCAACTACTGTGAC	GATGGCGTTCAGTTTCGTG	153
*INHA*	GCACCCTCCCAGTTTCATCT	GGTTGGGCACCATCTCATACT	230
*INHBA*	GCAGTCGCACAGACCTTTCCT	CTCACAGTAGTTGGCGTGGTAGC	196
*INHBB*	CCTCATCGGCTGGAACGACTGG	TGGACATGGTGCTCAGCTTGGTG	114
*FSHR*	GAAGAAAGCAGGTGGATGGA	GGCAGAGGAAAACTCCGTTA	126
*LHR*	GACACTAATTGCCACATCATCCT	GTGTCTTGGGTAAGCAGAAACC	203
*TGFBR3*	ACTGTTGCCCCACCATAGAG	CCTGGAAATCTTAGCCCTCA	103
*ACTB*	CATCGGCAATGAGCGGTTCC	CCGTGTTGGCGTAGAGGTCC	145

## Supplementary Materials

Supplementary materials can be found at http://www.mdpi.com/1422-0067/19/7/2028/s1, Figure S1: MT2 in the pshRNA-scramble (control) and pshRNA-MT2 group detected by the Western blot, Figure S2: ACTB in the pshRNA-scramble (control) and pshRNA-MT2 group detected by the Western blot, Supplementary tables: Effects of Effect of MT2 silencing and melatonin treatment on related genes expression and hormone secretions. Raw data for Ct value of 14 target genes, 1 reference gene, and the level of progesterone, estradiol, inhibin B and activin B in four experimental group: pshRNA-scramble (control), pshRNA-MT2 group, pshRNA-MT2 plus melatonin group and melatonin group.

## References

[1] Edson M.A., Nagaraja A.K., Matzuk M.M. (2009). The mammalian ovary from genesis to revelation. Endocr. Rev..

[2] Matsuda F., Inoue N., Manabe N., Ohkura S. (2012). Follicular growth and atresia in mammalian ovaries: Regulation by survival and death of granulosa cells. J. Reprod..

[3] Kaipia A., Hsueh A.J. (1997). Regulation of ovarian follicle atresia. Annu. Rev. Physiol..

[4] Nilsson E., Skinner M.K. (2001). Cellular interactions that control primordial follicle development and folliculogenesis. J. Soc. Gynecol. Investig..

[5] Richards J.S. (2001). Perspective: The ovarian follicle—A perspective in 2001. Endocrinology.

[6] Vitt U.A., Hsueh A.J. (2001). Stage-dependent role of growth differentiation factor-9 in ovarian follicle development. Mol. Cell. Endocrinol..

[7] Monget P., Fabre S., Mulsant P., Lecerf F., Elsen J.M., Mazerbourg S., Pisselet C., Monniaux D. (2002). Regulation of ovarian folliculogenesis by IGF and BMP system in domestic animals. Domest. Anim. Endocrinol..

[8] Orisaka M., Mizutani T., Tajima K., Orisaka S., Shukunami K., Miyamoto K., Kotsuji F. (2006). Effects of ovarian theca cells on granulosa cell differentiation during gonadotropin-independent follicular growth in cattle. Mol. Reprod. Dev..

[9] Zhen Y.H., Wang L., Riaz H., Wu J.B., Yuan Y.F., Han L., Wang Y.L., Zhao Y., Dan Y., Huo LJ. (2014). Knockdown of CEBPβ by RNAi in porcine granulosa cells resulted in S phase cell cycle arrest and decreased progesterone and estradiol synthesis. J. Steroid. Biochem. Mol. Biol..

[10] Jiang J.Y., Cheung C.K., Wang Y., Tsang B.K. (2003). Regulation of cell death and cell survival gene expression during ovarian follicular development and atresia. Front. Biosci..

[11] Choi J., Jo M., Lee E., Choi D. (2011). Induction of apoptotic cell death via accumulation of autophagosomes in rat granulose cells. Fertil. Steril..

[12] Forde N., Mihm M., Canty M.J., Zielak A.E., Baker P.J., Park S., Lonergan P., Smith G.W., Coussens P.M., Ireland J.J. (2008). Differential expression of signal transduction factors in ovarian follicle development: A functional role for betaglycan and FIBP in granulosa cells in cattle. Physiol. Genom..

[13] Mizutani T., Ishikane S., Kawabe S., Umezawa A., Miyamoto K. (2015). Transcriptional regulation of genes related to progesterone production. Endocr. J..

[14] Stocco D.M. (2001). StAR protein and the regulation of steroid hormone biosynthesis. Annu. Rev. Physiol..

[15] Matti N., Irving-Rodgers H.F., Hatzirodos N., Sullivan T.R., Rodgers R.J. (2010). Differential expression of focimatrix and steroidogenic enzymes before size deviation during waves of follicular development in bovine ovarian follicles. Mol. Cell. Endocrinol..

[16] Slominski A.T., Kim T.K., Shehabi H.Z., Semak I., Tang E.K., Nguyen M.N., Benson H.A., Korik E., Janjetovic Z., Chen J. (2012). In vivo evidence for a novel pathway of vitamin D_3_metabolism initiated by P450scc and modified by CYP27B1. FASEB J..

[17] Slominski A.T., Kim T.K., Chen J., Nguyen M.N., Li W., Yates C.R., Sweatman T., Janjetovic Z., Tuckey R.C. (2012). Cytochrome P450scc-dependent metabolism of 7-dehydrocholesterol in placenta and epidermal keratinocytes. Int. J. Biochem. Cell Biol..

[18] Slominski A.T., Li W., Kim T.K., Semak I., Wang J., Zjawiony J.K., Tuckey R.C. (2015). Novel activities of CYP11A1 and their potential physiological significance. J. Steroid Biochem. Mol. Biol..

[19] Ireland J.J., Roche J.F. (1982). Development of antral follicles in cattle after prostaglandin-induced luteolysis: Changes in serum hormones, steroids in follicular fluid, and gonadotropin receptors. Endocrinology.

[20] Ireland J.J., Roche J.F. (1983). Development of nonovulatory antral follicles inheifers: Changes in steroids in follicular fluid and receptors for gonadotropins. Endocrinology.

[21] Dubocovich M.L., Markowska M. (2005). Functional MT1 and MT2 melatonin receptors in mammals. Endocrine.

[22] Tamura H., Nakamura Y., Korkmaz A., Manchester L.C., Tan D.X., Sugino N., Reiter R.J. (2009). Melatonin and the ovary: Physiological and pathophysiological implications. Fertil. Steril..

[23] Tamura H., Takasaki A., Taketani T., Tanabe M., Kizuka F., Lee L., Tamura I., Maekawa R., Asada H., Yamagata Y. (2013). Melatonin as a free radical scavenger in the ovarian follicle. Endocr. J..

[24] Tian X., Wang F., He C., Zhang L., Tan D., Reiter R.J., Xu J., Ji P., Liu G. (2014). Beneficial effects of melatonin on bovine oocytes maturation: A mechanistic approach. J. Pineal Res..

[25] Wang S.J., Liu W.J., Wu C.J., Ma F.H., Ahmad S., Liu B.R., Han L., Jiang X.P., Zhang S.J., Yang L.G. (2012). Melatonin suppresses apoptosis and stimulates progesterone production by bovine granulosa cells via its receptors (MT1 and MT2). Theriogenology.

[26] Wang S., Liu B., Liu W., Xiao Y., Zhang H., Yang L. (2017). The effects of melatonin on bovine uniparental embryos development in vitro and the hormone secretion of COCs. Peer J..

[27] Wang S.J., Liu W.J., Wang L.K., Pang X.S., Yang L.G. (2017). The role of Melatonin receptor MTNR1A in the action of Melatonin on bovine granulosa cells. Mol. Reprod..

[28] Slominski R.M., Reiter R.J., Schlabritz-Loutsevitch N., Ostrom R.S., Slominski A.T. (2012). Melatonin membrane receptors in peripheral tissues: Distribution and functions. Mol. Cell. Endocrinol..

[29] Suofu Y., Li W., Jean-Alphonse F.G., Jia J., Khattar N.K., Li J., Baranov S.V., Leronni D., Mihalik A.C., He Y. (2017). Dual role of mitochondria in producing melatonin and driving GPCR signaling to block cytochrome c release. Proc. Natl. Acad. Sci. USA.

[30] Slominski A.T., Zmijewski M.A., Semak I., Kim T.K., Janjetovic Z., Slominski R.M., Zmijewski J.W. (2017). Melatonin, mitochondria, and the skin. Cell. Mol. Life Sci..

[31] Slominski A.T., Hardeland R., Zmijewski M.A., Slominski R.M., Reiter R.J., Paus R. (2018). Melatonin: A Cutaneous Perspective on its Production, Metabolism, and Functions. J. Investig. Dermatol..

[32] Nakamura Y., Tamura H., Takayama H., Kato H. (2003). Increased endogenous level of melatonin in preovulatory human follicles does not directly influence progesterone production. Fertil. Steril..

[33] Liu W.J., Wang S.J., Zhou J.X., Pang X.S., Wang L.K. (2018). RNAi-mediated knockdown of MTNR1B without disrupting the effects of melatonin on apoptosis and cell cycle in bovine granulose cells. Peer J..

[34] Matos M.H., Lima-Verde I.B., Luque M.C., Maia J.E., Silva J.R., Celestino J.J., Martins F.S., Bao S.N., Lucci C.M., Figueiredo J.R. (2007). Essential role of follicle stimulating hormone in the maintenance of caprine preantral follicle viability in vitro. Zygote.

[35] Saraiva M.V., Rossetto R., Brito I.R., Celestino J.J., Silva C.M., Faustino L.R., Almeida A.P., Bruno J.B., Magalhães D.M., Matos M.H. (2010). Dynamic medium produces caprine embryo from preantral follicles grown in vitro. Reprod. Sci..

[36] Misztal T., Romanowicz K. (2005). Effective stimulation of daily LH secretion by the combined treatment with melatonin and naloxone in luteal-phase ewes. Acta Neurobiol. Exp. (Wars).

[37] He C.J., Ma T., Shi J.M., Zhang Z.Z., Wang J., Zhu K., Li Y., Yang M., Song Y., Liu G. (2016). Melatonin and its receptor MT1 are involved in the downstream reaction to luteinizing hormone and participate in the regulation of luteinization in different species. J. Pineal Res..

[38] Saraiva M.V., Celestino J.J., Araújo V.R., Chaves R.N., Almeida A.P., Lima-Verde I.B., Duarte A.B., Silva G.M., Martins F.S., Bruno J.B. (2011). Expression of follicle-stimulating hormone receptor (FSHR) in goat ovarian follicles and the impact of sequential culture medium on in vitro development of caprine preantral follicles. Zygote.

[39] Barros V.R., Cavalcante A.Y., Macedo T.J., Barberino R.S., Lins T.L., Gouveia B.B., Menezes V.G., Queiroz M.A., Araújo V.R., Palheta R.C. (2013). Immunolocalization of melatonin and follicle-stimulating hormone receptors in caprine ovaries and their effects during in vitro development of isolated pre-antral follicles. Reprod. Domest. Anim..

[40] Woo M.M., Tai C.J., Kang S.K., Nathwani P.S., Pang S.F., Leung P.C. (2001). Direct action of melatonin in human granulosa-luteal cells. J. Clin. Endocrinol. Metab..

[41] Pan Z., Zhang J., Lin F., Ma X., Wang X., Liu H. (2012). Expression profiles of key candidate genes involved in steroidogenesis during follicular atresia in the pig ovary. Mol. Biol. Rep..

[42] Tanavde V.S., Maitra A. (2003). In vitro modulation of steroidogenesis and gene expression by melatonin: A study with porcineantral follicles. Endocr. Res..

[43] Swan C.L., Agostini M.C., Bartlewski P.M., Feyles V., Urban R.J., Chedrese P.J. (2002). Effects on progesterone synthesis in a stable porcine granulosa cell line: Control of transcriptional activity of the cytochrome P450Side-chain cleavage gene. Biol. Reprod..

[44] Miller W.L. (2007). Steroidogenic acute regulatory protein (StAR), a novel mitochon- drial cholesterol transporter. Biochim. Biophys. Acta.

[45] Christenson L.K., Stouffer R.L., Strauss J.F. (2001). Quantitative analysis of the hormone-induced hyperacetylation of histoneH3associated with the steroido-genic acute regulatory protein gene promoter. J. Biol. Chem..

[46] Park E.S., Lind A.K., Dahm-Kahler P., Brannstrom M., Carletti M.Z., Christenson L.K., Curry T.E., Jo M. (2010). RUNX2 transcription factor regulates gene expression in luteinizing granulosa cells of rat ovaries. Mol. Endocrinol..

[47] Papamentzelopoulou M., Mavrogianni D., Dinopoulou V., Theofanakis H., Malamas F., Marinopoulos S., Bletsa R., Anagnostou E., Kallianidis K., Loutradis D. (2012). Detection of RUNX2 gene expression in cumulus cells in women undergoing controlled ovarian stimulation. Reprod. Biol. Endocrinol..

[48] Shimasaki S., Moore R.K., Otsuka F., Erickson G.F. (2004). The bone morpho-genetic protein system in mammalian reproduction. Endocr. Rev..

[49] Otsuka F. (2013). Multifunctional bone morphogenetic protein system in endocrinol-ogy. Acta Med. Okayama.

[50] Erickson G.F., Shimasaki S. (2003). The spatiotemporal expression pattern of the bonemorphogenetic protein family in rat ovary cell types during the estrous cycle. Reprod. Biol. Endocrinol..

[51] Shimasaki S., Moore R.K., Erickson G.F., Otsuka F. (2003). The role of bonemorphogenetic proteins in ovarian function. Reprod. Suppl..

[52] Nakamura E., Otsuka F., Terasaka T., Inagaki K., Hosoya T., Tsukamoto-Yamauchi N., Toma K., Makino H. (2014). Melatonin counteracts BMP-6 regulation of steroidogenesis by rat granulosa cells. J. Steroid. Biochem. Mol. Biol..

[53] Otsuka F., Moore R.K., Shimasaki S. (2001). Biological function and cellular mecha-nism of bone morphogenetic protein-6 in the ovary. J. Biol. Chem..

[54] Jimenez-Krassel F., Winn M.E., Burns D., Ireland J.L., Ireland J.J. (2003). Evidence for a negative intrafollicular role for inhibin in regulation of estradiol production by granulosa cells. Endocrinology.

[55] Young J.M., Henderson S., Souza C., Ludlow H., Groome N., McNeilly A.S. (2012). Activin B is produced early in antral follicular development and suppresses thecal androgen production. Reproduction.

[56] Chang H.M., Cheng J.C., Huang H.F., Shi F.T., Leung P.C. (2015). Activin A, B and AB decrease progesterone production by down-regulating StAR in human granulosa cells. Mol. Cell. Endocrinol..

[57] Kaneko H., Noguchi J., Kikuchi K., Todoroki J., Hasegawa Y. (2002). Alterations in peripheral concentrations of inhibinAin cattle studied using a time-resolved immunofluorometric assay: Relationship with estradiol and follicle-stimulating hormone in various reproductive conditions. Biol. Reprod..

[58] Thackray V.G., Mellon P.L., Coss D. (2010). Hormones in synergy: Regulation of the pituitary gonadotropin genes. Mol. Cell. Endocrinol..

[59] Han L., Wu C., Riaz H., Bai L., Chen J., Zhen Y., Guo A., Yang L. (2013). Characterization of the mechanism of inhibin α-subunit gene in mouse anterior pituitary cells by RNA interference. PLoS ONE.

[60] Takedomi T., Kishi H., Medan M.S., Aoyagi Y., Konishi M., Itoh T., Yazawa S., Watanabe G., Taya K. (2005). Active immunization against inhibin improves superovulatory response to exogenous FSH in cattle. J. Reprod. Dev..

[61] Han L., Mao D.G., Zhang D.K., Liang A.X., Fang M., Moaeen-ud-Din M., Yang L.G. (2008). Development and evaluation of a novel DNA vaccine expressing inhibin alpha (1–32) fragment for improving the fertility in rats and sheep. Anim. Reprod. Sci..

[62] Dan X., Liu X., Han Y., Liu Q., Yang L. (2016). Effect of the novel DNA vaccine fusing inhibin α (1–32) and the RF-amide related peptide-3 genes onimmune response, hormone levels and fertility in Tan sheep. Anim. Reprod. Sci..

[63] Wang F., Tian X., Zhang L., Gao C., He C., Fu Y., Ji P., Li Y., Li N., Liu G. (2014). Beneficial effects of melatonin on in vitro bovine embryonic development are mediated by melatonin receptor 1. J. Pineal Res..

[64] Tian X., Wang F., Zhang L., He C., Ji P., Wang J., Zhang Z., Lv D., Abulizi W., Wang X. (2017). Beneficial Effects of Melatonin on the In Vitro Maturation of Sheep Oocytes and Its Relation to Melatonin Receptors. Int. J. Mol. Sci..

[65] Li R., Albertini D.F. (2013). The road to maturation: Somatic cell interaction and self-organization of the mammalian oocyte. Nat. Rev. Mol. Cell Biol..

[66] Jackson-Grusby L., Beard C., Possemato R., Tudor M., Fambrough D., Csankovszki G., Dausman J., Lee P., Wilson C., Lander E. (2001). Loss of genomic methylation causes p53-dependent apoptosis and epigenetic deregulation. Nat. Genet..

[67] Slominski A.T., Kim T.K., Takeda Y., Janjetovic Z., Brozyna A.A., Skobowiat C., Wang J., Postlethwaite A., Li W., Tuckey R.C. (2014). RORα and RORγ are expressed in human skin and serve as receptors for endogenously produced noncalcemic 20-hydroxy and 20,23-dihydroxyvitamin D. FASEB J..

[68] Steinhilber D., Brungs M., Werz O., Wiesenberg I., Danielsson C., Kahlen J.P., Nayeri S., Schräder M., Carlberg C. (1995). The nuclear receptor for melatonin represses 5-lipoxygenase gene expression in human B lymphocytes. J. Biol. Chem..

[69] Slominski A.T., Zmijewski M.A., Jetten A.M. (2016). RORα is not a receptor for melatonin (response to DOI 10.1002/bies.201600018). Bioessays.

[70] Prendergast B.J. (2010). MT1 melatonin receptors mediate somatic, behavioral, and reproductive neuroendocrine responses to photoperiod and melatonin in Siberian hamsters (*Phodopus sungorus*). Endocrinol.

[71] He C., Wang J., Zhang Z., Yang M., Li Y., Tian X., Ma T., Tao J., Zhu K., Song Y. (2016). Mitochondria Synthesize Melatonin to Ameliorate Its Function and Improve Mice Oocyte’s Quality underin Vitro Conditions. Int. J. Mol. Sci..

[72] Jia Y., Yang M., Zhu K., Wang L., Song Y., Wang J., Qin W., Xu Z., Chen Y., Liu G. (2016). Melatonin implantation improved the egg-laying rate and quality in hens past their peak egg-laying age. Sci. Rep..

[73] Espino J., Ortiz Á., Bejarano I., Lozano G.M., Monllor F., García J.F., Rodríguez A.B., Pariente J.A. (2011). Melatonin protects human spermatozoa from apoptosis via melatonin receptor- and extracellular signal-regulated kinase-mediated pathways. Fertil. Steril..

[74] Espino J., Rodríguez A.B., Pariente J.A. (2013). The inhibition of TNF-α-induced leucocyte apoptosis by melatonin involves membrane receptor MT1/MT2 interaction. J. Pineal Res..

[75] Radogna F., Cristofanon S., Paternoster L., D’Alessio M., De Nicola M., Cerella C., Dicato M., Diederich M., Ghibelli L. (2008). Melatonin antagonizes the intrinsic pathway of apoptosis via mitochondrial targeting of Bcl-2. J. Pineal Res..

[76] Livak K.J., Schmittgen T.D. (2001). Analysis of relative gene expression data using real-time quantitative PCR and the 2(-Delta Delta C(T)) Method. Methods.

