# Supplementary material for: The Mechanism of Melatonin and Its Receptor MT2 Involved in the Development of Bovine Granulosa Cells
Source: Int J Mol Sci. 2018 Jul 12;19(7):2028. doi: 10.3390/ijms19072028 (PMC6073438; doi:10.3390/ijms19072028)
Supplement: Supplementary file 1 [file ijms-19-02028-s001.zip › ijms-304284-supplementary materials/supplementary figures.docx]

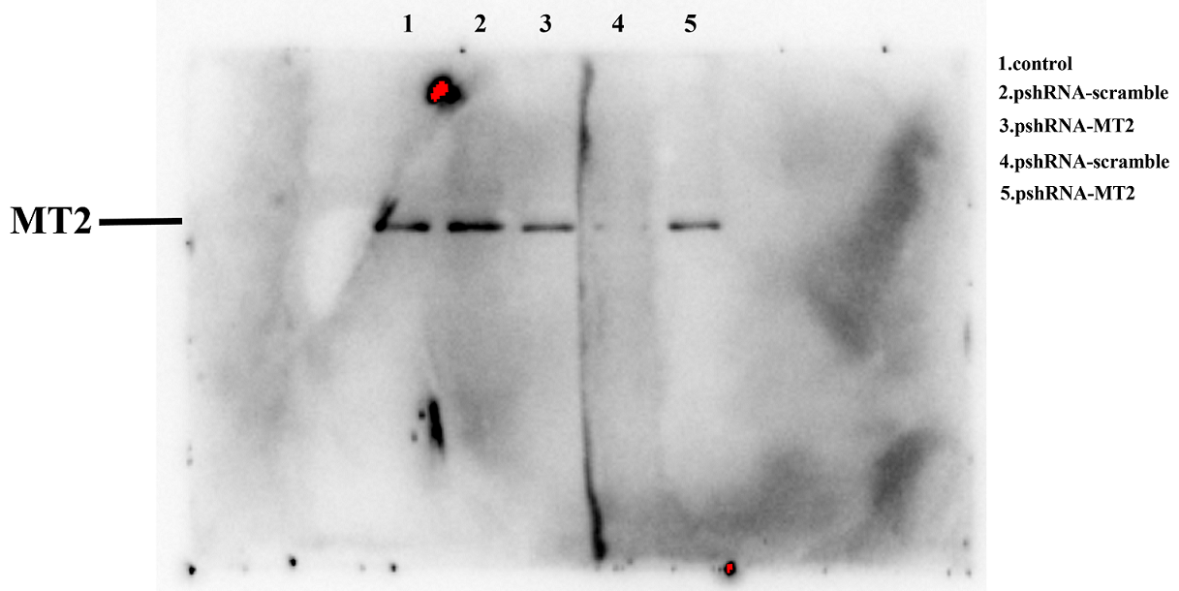


**Figure S1.** MT2 in the pshRNA-scramble (control) and pshRNA-MT2 group detected by the Western blot.


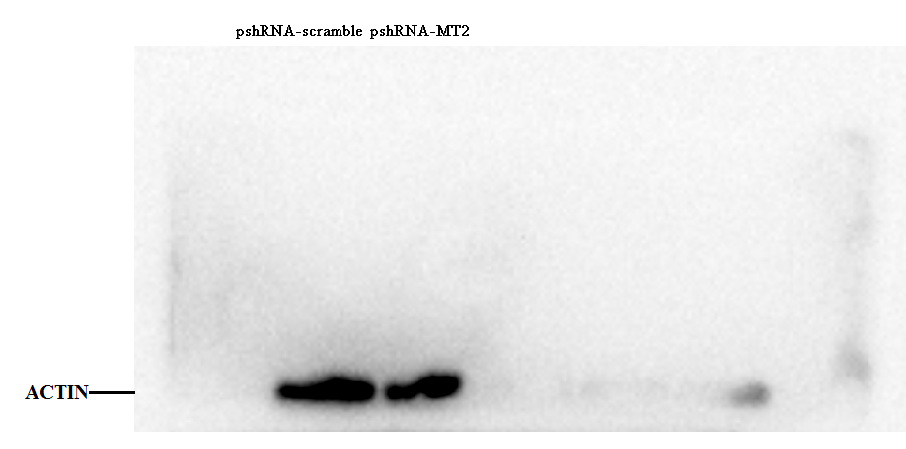


**Figure S2.** ACTB in the pshRNA-scramble (control) and pshRNA-MT2 group detected by the Western blot.
